# The Quality and Starch Digestibility of Multi-Grain Noodles Are Regulated by the Additive Amount of Dendrobium Officinale

**DOI:** 10.3390/foods14030413

**Published:** 2025-01-27

**Authors:** Xinyu Zhang, Pai Peng, Qianying Ma, Shance Niu, Shande Duan, Yimeng Zhang, Xinzhong Hu, Xiaolong Wang

**Affiliations:** 1 College of Food Engineering and Nutritional Science, Shaanxi Normal University, Xi’an 710119, China; xinyzhang0314@163.com (X.Z.); pengpai1015@126.com (P.P.); qianying2021@126.com (Q.M.); 17791056990@163.com (Y.Z.); hxinzhong@snnu.edu.cn (X.H.); 2 College of Horticulture, Hebei Agricultural University, Baoding 071001, China; duansd940603@163.com; 3 State Key Laboratory of Crop Improvement and Regulation in North China, Hebei Agricultural University, Baoding 071001, China

**Keywords:** dendrobium officinale (DO), multi-grain noodles, noodle quality, starch digestibility

## Abstract

Dendrobium officinale (DO) is a well-known medicinal and edible plant, yet its impact on the quality of noodles has been infrequently reported. In this study, DO was incorporated into multi-grain flour in varying proportions (0, 2, 4, 6, 8%) to prepare noodles, and their quality was assessed. The percentage increase in DO decreased the cooking loss, whiteness, appearance, and taste of the noodles while simultaneously enhancing their water absorption, adhesiveness, smoothness, and starch digestion resistance. Lower supplemental levels of DO (2–4%) facilitated the water absorption of protein and the formation of a dense and extensive protein network surrounding the partially gelatinized starch, which was characterized by higher relative crystallinity. The highest sensory score (77.4) and greatest content of slowly digestible starch content (38%) were observed in the noodles containing 4% DO. Conversely, higher percentages of DO (6–8%) diluted and compromised the protein network in the cooked noodles, leading to water migration from protein to starch. The excessive polysaccharides from DO tended to complex with fully gelatinized starch, promoting starch aggregation and interactions between starch and non-starch components. This ultimately resulted in the highest adhesiveness and resistant starch content (34%) in the cooked noodles with 8% DO. These findings provide a reference for enhancing noodle quality by regulating the amount of DO added, thereby promoting the application of DO in cereal-based food products.

## 1. Introduction

Wheat noodles are a traditional staple food in China, and the gluten protein, accounting for 75–85% of the total protein in wheat flour, gives unique viscoelasticity to different wheat noodles [1]. However, due to the high starch content in refined wheat flour, the long-term consumption of wheat noodles at high levels may lead to chronic diseases such as hyperglycemia and diabetes. In contrast, buckwheat, oat, and other coarse grains are rich in dietary fiber, polyphenols, flavonoids, and other bioactive substances, and the addition of them to noodles leads to improved nutritional quality and reduced starch digestibility. However, because of the lack of gluten protein, the amount of oat or buckwheat flour added to the noodle formula has been limited to ~20% (dry base), with the high percentage of coarse grains usually leading to poor performance in cooking loss, texture, and edibility quality [2,3]. In order to increase the amount of coarse grains added and balance the nutritional quality and texture properties of multi-grain noodles, heat–moisture treatments of multi-grain flour (microwaving, autoclaving, extruding, etc.) [4], vacuum mixing [5], natural fermentation [6], and quality improvers (hydrophilic colloid, phosphate, gluten, etc.) have been commonly used in the making of multi-grain noodles [7].

A variety of polysaccharides, such as guar gum, xanthan gum, artemisia gum, sodium alginate, carboxymethyl cellulose, and carrageenan, have been widely used in noodle preparation due to their excellent gelling ability, water solubility, water retention, and thickening properties. They can enhance the water absorption and gelatinization of flour, interact with protein to form a denser network structure, improve rehydration and viscoelasticity, and reduce the breakage rate and cooking time of noodles [8]. Liu et al. [9] discovered that tremella polysaccharide can effectively inhibit the migration of free water in fresh and wet noodles, which contributed to a more compact noodle microstructure with a well-organized gluten network, finally leading to the improved water retention and storage stability of noodles. Li et al. [10] found the protein–polysaccharide extracted from Auricularia auricula-judae mushrooms could improve the stability and edible quality of noodles by promoting the cross-linking of the protein molecular network and enhancing the dense microstructure of dough. Carrageenan has also been found to significantly improve the microstructural compactness, hardness, springiness, and chewiness of cooked noodles [11]. Zhou et al. [12] reported that the addition of <4% konjac glucomannan to wheat noodles increased the cooking yield, hardness, and springiness of cooked noodles.

Dendrobium officinale (DO), a well-known medicinal and edible plant of Dendrobium [13], was officially included in the catalog of food and Chinese medicinal materials in November 2023. It contains a variety of chemical components such as polysaccharides, alkaloids, bibenzylates, flavonoids, amino acids, and trace elements, among which the polysaccharide, a linear glucomannan consisting of β-1,4-linked mannose [14], is the most important active component [15]. The DO polysaccharide has great water retention, water absorption, and gel properties that are similar to the polysaccharides used in noodle quality improvement. However, to date, the studies on DO have mainly focused on its antioxidant, anti-tumor, anti-fatigue, body immunity improvement, and pharmacological activity with respect to diabetes. Although some new food products containing DO, such as soft drinks, wine, and crackers, have been effectively developed [16] and fresh Dendrobium officinale juice was applied in the preparation of healthy noodles [17], the effect and underlying mechanism of DO addition on the quality and digestive properties of multi-grain noodles have not been reported. We speculate that the complex interactions between DO and starch or protein during the processing and cooking of multi-grain noodles have the potential to improve the texture performance and digestion resistance of noodles. Therefore, in this study, DO was added to multi-grain noodles to explore its effects on the quality and digestive characteristics of noodles and the underlying mechanism. The findings will provide theoretical and technical references for the application of DO in the production of multi-grain-based staple foods.

## 2. Materials and Methods

### 2.1. Materials

High-gluten wheat flour was obtained from Xi’an Aiju Grain and Oil Co., Ltd. (Xi’an, China). Oat flour was obtained from Inner Mongolia Yinshan Youmai Food Co., Ltd. (Ulanqab, China). Sweet buckwheat flour was obtained from Dingbian Saixue Flour Co., Ltd. (Xi’an, China). The dendrobium officinale variety named Muge 1 was obtained from the College of Horticulture, Hebei Agricultural University (Baoding, China). The basic chemical compositions of the fours are listed in Table 1. All the chemicals and solvents purchased from Tianjin Damao Chemical Co., Ltd. (Tianjin, China) were of analytical grade.

### 2.2. Preparation of Dendrobium Officinale Powder

Fresh DO stems were dried at 50 °C for 44 h in a hot air-drying oven (Model 101, Shenzhen Zhongxing Environmental Engineering Technology Co., Ltd., Shenzhen, China), ground into power with a grinder (Model H8422 168/140, Hebei Deke Machinery Technology Co., Ltd., Baoding, China), and then sieved through 80 mesh.

### 2.3. Preparation of Multi-Grain Noodles

Oat flour, common buckwheat flour, and wheat flour were mixed in a 3:1:6 ratio to create the multi-grain flour. A total of 100 g of this multi-grain flour was combined with varying amounts of DO (0%, 2%, 4%, 6%, or 8%), along with 36% water, utilizing a flour mixer (Model KVL8300S. Keywgod, London, UK). The resulting mixture was then wrapped in plastic wrap and allowed to rest for 20 min at room temperature. Subsequently, the dough was processed through an experimental noodle machine (JMTD 168/140, Beijing Dongfujiuheng Instrument Co., Ltd., Beijing, China) with a decreasing roll gap set at 2.0, 1.5, and 1.0 mm. The sheet of the dough was then cut into fresh noodles measuring 2.0 mm in width and 1.0 mm in thickness. The freshly prepared noodles were evaluated immediately for cooking and sensory quality. Additionally, surplus noodles were lyophilized and stored at −20 °C for further analysis.

### 2.4. Cooking Quality Test of Noodles

Noodles of 30 g were cooked in 500 mL of boiling water. The cooking properties of the noodles, including optimum cooking time, cooking loss, and water absorption, were measured according to the method described by Guo et al. [18]. When determining cooking loss and water absorption, all operations were conducted at room temperature (25 °C), except for the noodle and soup drying process, which was performed at an oven temperature of 105 °C.

### 2.5. Texture Profile Analysis (TPA) of Noodles

The textural properties of all samples were measured using a TA.XT.Plus Texture Analyzer (Stable Micro Systems, Godalming, UK). A 50 g portion of the fresh noodles was cooked in a stainless steel basin containing 500 mL of boiling water. The cooked noodles were taken out at the optimal cooking time and cooled in cold water for 1 min for further evaluation. The samples were compressed twice to 75% of their original height using a cylindrical probe (P/36R). The speeds before, during, and after the test were 2, 2, and 1 mm/s, respectively, with a trigger force of 5 g [19]. All samples were measured in triplicate to determine their hardness, adhesiveness, springiness, and chewiness.

### 2.6. Sensory Evaluation of Noodles

A sensory evaluation of the noodles was conducted in accordance with the Chinese business standard SB/T 10137-93 [20]. A sensory evaluation team composed of eight professionally trained noodle researchers, consisting of four men and four women, was selected to assess and score various attributes of the cooked noodles including color, appearance (surface flatness), palatability (softness or hardness), toughness, stickiness, smoothness, and taste. The team underwent 2 h of training to ensure that the results were objective and aligned with the sensory descriptions. A detailed explanation of the scoring criteria is provided in Supplementary Material Table S1.

### 2.7. Gelatinization Degree Measurement of Noodles

According to the method described by Birch [21], 0.2 g of lyophilized cooked noodle powder was suspended in 98 mL of distilled water, followed by the addition of 2 mL of 10 M KOH solution. The mixture was magnetically stirred for 5 min and then centrifuged at 3500 rpm for 30 min. Subsequently, 0.2 mL of the supernatant was collected and mixed with 0.2 mL of 0.2 M HCl solution, 15 mL of distilled water, and 0.2 mL of iodine solution (composed of 1 g of iodine and 4 g of potassium iodide in a total volume of 100 mL) to measure the absorbance *A*1 at 600 nm. In a similar manner, a separate sample of 0.5 g was suspended in 95 mL of distilled water before the addition of 5 mL of 10 M KOH solution. After magnetic stirring for 5 min, this sample was also centrifuged at 3500 rpm for 30 min. Then, 0.2 mL of the supernatant was taken and mixed with 0.2 mL of 0.5 M HCl solution, 15 mL of distilled water, and 0.2 mL of iodine solution to measure the absorbance *A*2 at 600 nm. The gelatinization degree of the cooked noodles was calculated as follows:

(1) $$\mathrm{Gelatinization}\;\mathrm{degree}\;(\%)=\frac{A1}{A2}\times 100\%$$

### 2.8. Determination of In Vitro Digestion Characteristics of Noodle Starch

According to the method described by Chen [22], lyophilized powders of cooked noodles containing 50 mg of starch were hydrolyzed using 4 mL of porcine pancreatic enzyme solution (0.03 g/mL, 4 × USP, P1750-100G, Shanghai Hengdu Biotechnology Co., Ltd., Shanghai, China) and 1 mL of glucoamylase solution (0.025 mL/mL, 1 × 10^5^ U/mL, A107823-10ML, Shanghai Aladdin Biochemical Technology Co., Ltd., Shanghai, China). The glucose concentration at each specific time point (0, 10, 20, 30, 60, 90, 120, and 180 min) was determined using a biosensor (S-10, Shenzhen Sieman Technology Co., Ltd., Shenzhen, China) to calculate the contents of rapidly digestible starch (RDS), slowly digestible starch (SDS), and resistant starch (RS).

The hydrolysis index (HI) was calculated by considering the area under the hydrolysis curve, expressed as a percentage ratio of the area under the curve for the test food compared to the standard food (white bread). The estimated glycemic index (eGI) was calculated using the values obtained for HI, substituting them into the equation provided by Goni et al. [23]. The HI and eGI values for cooked noodles were calculated as follows:

(2) $$\mathrm{HI}=\frac{S}{SW}\times 100$$

(3) eGI=39.71+0.549×HI

where S represents the area under the hydrolysis curve for the test food, while SW denotes the area under the hydrolysis curve for the standard food.

### 2.9. X-Ray Diffraction (XRD) Analysis

The lyophilized noodle powders were analyzed using an X-ray diffractometer (Rigaku Dmax/2550, Shibuya, Japan) [24]. The noodle powders were flattened, and the scanning was conducted over a range of 4 to 40° at a scanning rate of 2°/min with a step size of 0.02°. Changes in the starch crystal structure were identified by the shape of the diffraction peaks. Two independent measurements were performed for each sample.

### 2.10. Fourier Transform Infrared Spectroscopy (FTIR) Analysis

The protein secondary structure and the interactions of starch, protein, and lipid in the noodles were analyzed by using a Vertex 70 FTIR spectrometer (Bruker., Ltd. Berlin, Germany). The lyophilized powders of cooked noodles and spectral-grade KBr (Kermal, SP, CAS: 7758-02-3) were dried at 40 °C for 8–12 h, mixed in a ratio of 1:100, and the ground in an agate mortar before being pressed into 20 mg thin slices at 11 LBS. The background was scanned 64 times in the range of 400–4000 cm^−1^ at a resolution of 4 cm^−1^ [25]. The corresponding regions of different protein secondary structure types in the amide I region (1600–1700 cm^−1^) are as follows: 1612–1618 cm^−1^, 1625–1635 cm^−1^, and 1685–1695 cm^−1^ correspond to β-sheets, 1640–1655 cm^−1^ corresponds to random coils, 1658–1665 cm^−1^ corresponds to α-helices, and 1670–1680 cm^−1^ corresponds to β-turns.

### 2.11. Nuclear Magnetic Resonance (NMR) Analysis

The carbon skeleton structure of starch was determined using ^13^C CP/MAS NMR (JNM-ECZ400R/S1, JEOL Ltd., Tokyo, Japan). The ^13^C CP/MAS NMR spectra were obtained by rotating the sample tube at 1200 Hz with a double-resonance H/X CP-MAS 4 mm probe [26].

### 2.12. Size-Exclusion High-Performance Liquid Chromatography (SE-HPLC) Analysis of Noodle Proteins

Molecular size distributions of noodle proteins were determined using an Ultimate 3000 HPLC system (Thermo Scientific, Carlsbad, CA, USA) equipped with a TSK gel G4000SWXL (7.8 × 300 mm) column (Tosoh Corporation, Tokyo, Japan), following the method described by Jia [27]. The protein extraction chromatogram can be categorized into five components based on their molecular weights: the first three fractions (F1, F2, F3) correspond to the largest polymeric protein (LPP), the middle polymeric protein (MPP), and the smaller polymeric protein (SPP), respectively. The last two fractions (F4, F5) correspond to the larger and smaller monomeric proteins (LMP and SMP), respectively. The proportion of sodium dodecyl sulfate-unextractable proteins in the first two fractions was designated as UPP%. The peaks of extractable and unextractable LPP, MPP, SPP, LMP, and SMP were labeled as F1e and F1u, F2e and F2u, F3e and F3u, F4e and F4u, and F5e and F5u, respectively. The contents of the different protein components were calculated as follows:

(4) Total area=F1e+F1u+F2e+F2u+F3e+F3u+F4e+F4u+F5e+F5u

(5) $$\mathrm{LPP}\;(\%)=100\times \frac{F1e+F1u}{Total\;area}$$

(6) $$\mathrm{MPP}\;(\%)=100\times \frac{F2e+F2u}{Total\;area}$$

(7) $$\mathrm{SPP}\;(\%)=100\times \frac{F3e+F3u}{Total\;area}$$

(8) $$\mathrm{LMP}\;(\%)=100\times \frac{F4e+F4u}{Total\;area}$$

(9) $$\mathrm{SMP}\;(\%)=100\times \frac{F5e+F5u}{Total\;area}$$

(10) $$\mathrm{UPP}\;(\%)=100\times \frac{F1u+F2u}{Total\;area}$$

### 2.13. Microstructure Analysis by Confocal Laser Scanning Microscope (CLSM)

CLSM imaging was conducted using an Olympus FV1200 CLSM (Olympus, Tokyo, Japan) equipped with an inverted microscope (Olympus, Japan), following the previously established method [24]. The frozen noodles were sectioned into 12 μm slices using a freezing microtome (CM 1950 UV, Leica Microsystems, Wetzlar, Germany) and stained with 0.25% (*w*/*v*) 5-isothiocyanate fluorescein (FITC) (Sigma-F7250, ≥90%, CAS: 3326-32-7) and 0.025% (*w*/*v*) rhodamine B (Sigma-R6625, AR, CAS: 81-88-9) (Sigma Aldrich Corporation, St. Louis, MO, USA) for 10 min. Finally, three washes with deionized water were performed to remove any bound fluorescent dyes prior to the final observation.

### 2.14. Particle Size Determination

A laser particle size analyzer (LS13320, Beckman Coulter Instruments, Indianapolis, IN, USA) was utilized to determine the particle size distribution of lyophilized powders from the cooked noodles. The flour was dispersed in distilled water, and its particle size distribution was measured when the obscuration reached 7–13%. The parameters D10, D50, and D90 represent the particle sizes corresponding to the cumulative particle size distribution of the flour at 10%, 50%, and 90%, respectively, with D50 indicating the median diameter [28].

### 2.15. Determination of Water Migration and Distribution in Cooked Noodles

Referring to the method of Liu et al. [29], noodle samples with different DO contents were wrapped with PTFE tape and put into a nuclear magnetic tube with a diameter of 25 mm. Then, the prepared samples were analyzed in an NMI20-025V LF-NMR analyzer (Niumag Analytical Instrument Co., Ltd., Suzhou, China) equipped with a 0.3 ± 0.05-T permanent magnet.

### 2.16. Statistical Analysis

An analysis of variance (ANOVA) was conducted to assess significant differences using SPSS Version 25.0 software (IBM software, Chicago, IL, USA). Significant differences (*p* < 0.05) were determined using Duncan’s procedure. All figures were created using Origin 2019 (OriginLab Corporation, Northampton, MA, USA).

## 3. Results and Discussion

### 3.1. Quality Characteristics of the Noodles

#### 3.1.1. Cooking Quality

As shown in Table 2, the cooking time of the noodles increased continuously from 3.00 min to 3.75 min with a proportional increase in DO from 0% to 8%. This may be attributed to the fact that DO contains a significant amount of polysaccharides that compete with starch for water absorption [30]. Additionally, DO polysaccharides can wrap around starch granules, forming a physical barrier [31] that inhibits the water absorption and swelling of starch, thereby delaying starch gelatinization. The length of cooking time is positively correlated with the compactness of the noodles, which significantly influences water absorption, water mobility, protein denaturation, and starch gelatinization [32]. When the percentage of DO is between 2% and 4%, the compact gluten network in the noodles restricts water absorption and mobility, as well as starch gelatinization, thereby prolonging the cooking time. When the amount of DO increases from 6% to 8%, the excessive DO polysaccharides facilitate the extensive aggregation of partially gelatinized starch, resulting in a reduced porosity of the noodles and ultimately extending the cooking time. The increasing content of DO also leads to a significant rise in the water absorption of the noodles from 67.42% to 87.33%. In contrast, the cooking loss of the noodles decreases from 17.68% to 9.25%. Since DO is rich in polysaccharides with a high number of hydroxyl groups, which exhibit a stronger water-holding capacity than wheat protein and starch [33,34], it is likely that the increased polysaccharide content not only enhances the water-holding capacity of noodles but also promotes the formation of a macromolecular network (including polysaccharides and gluten proteins) that tightly encapsulates the starch granules. This combination leads to increased water absorption and decreased cooking loss.

#### 3.1.2. Textural Properties

**Figure 1:**
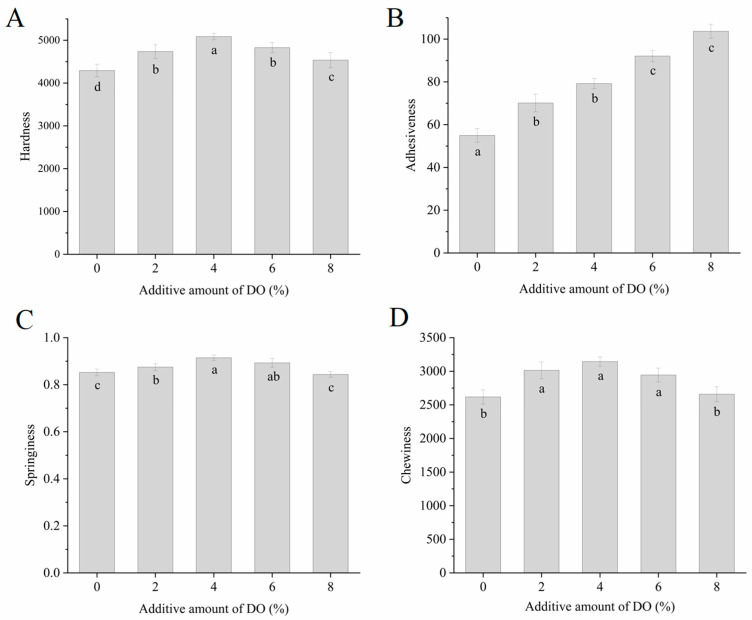
The texture quality of cooked multi-grain noodles. (**A**) The hardness of noodles; (**B**) the adhesiveness of noodles; (**C**) the springiness of noodles; (**D**) the chewiness of noodles. DO, Dendrobium officinale. Different small letters in the graphs indicate significant differences (*p* ≤ 0.05) among cooked noodles that differ in DO content.

Figure 1 illustrates the textural characteristics of the cooked multi-grain noodles with varying concentrations of DO. The hardness, adhesiveness, springiness, and chewiness of the cooked noodles increased consistently as the DO content rose from 0% to 4%. However, a further increase in DO from 4% to 8% resulted in a decrease in hardness, springiness, and chewiness, while adhesiveness increased. The textural changes observed in the cooked noodles with different DO levels may be attributed to the macromolecular interactions between gluten proteins and Dendrobium polysaccharides. A lower proportion of DO (2% and 4%) appears to promote the aggregation of wheat proteins and the formation of a more extensive and compact macromolecular network, likely involving gluten polymers and Dendrobium polysaccharides, which tightly encapsulate the partially gelatinized starch granules and significantly enhance the viscoelasticity of the cooked noodles. In this scenario, the increase in noodle adhesiveness is more closely related to the high viscosity of Dendrobium polysaccharides than to the degree of starch gelatinization. When the percentage of DO exceeds 4%, excessive Dendrobium polysaccharides and other components from DO likely inhibit the formation of the macromolecular network due to the dilution of gluten proteins [35] and completion for water absorption between gluten proteins and Dendrobium polysaccharides [36]. This combination results in poorer textural characteristics of the cooked noodles. Additionally, the increased adhesiveness of the cooked noodles at high DO levels may be attributed to the high viscosity of Dendrobium polysaccharides and the higher degree of starch gelatinization facilitated by the sparse gluten network surrounding the starch. Certain food gums exhibit similar effects on noodle textural characteristics. For instance, a small amount of carrageenan can enhance the hardness, elasticity, and chewability of low-sodium noodles, but excessive addition can lead to a deterioration in noodle texture [11]. The amount of konjac gum added is positively correlated with the hardness and viscosity of noodles; however, elasticity initially increases and then decreases, with maximum elasticity observed in noodles containing 3% konjac gum [12].

#### 3.1.3. Sensory Quality

The sensory qualities of the multi-grain noodles with varying amounts of DO addition are presented in Table 3. Generally, the percentage increase in DO resulted in a decline in the whiteness, appearance, and taste of the noodles while enhancing their stickiness and smoothness. The palatability and toughness of the cooked noodles increased as the DO concentration rose from 0% to 4%, but they decreased when the DO level exceeded 4%. This phenomenon can be attributed to the deep color and herbal flavor characteristic of DO; excessive amounts likely diminished the whiteness and overall taste of the noodles. Notably, the noodles with 4% DO achieved the highest sensory score of 77.4.

### 3.2. Starch Properties of the Noodles

#### 3.2.1. Gelatinization Property

**Figure 2:**
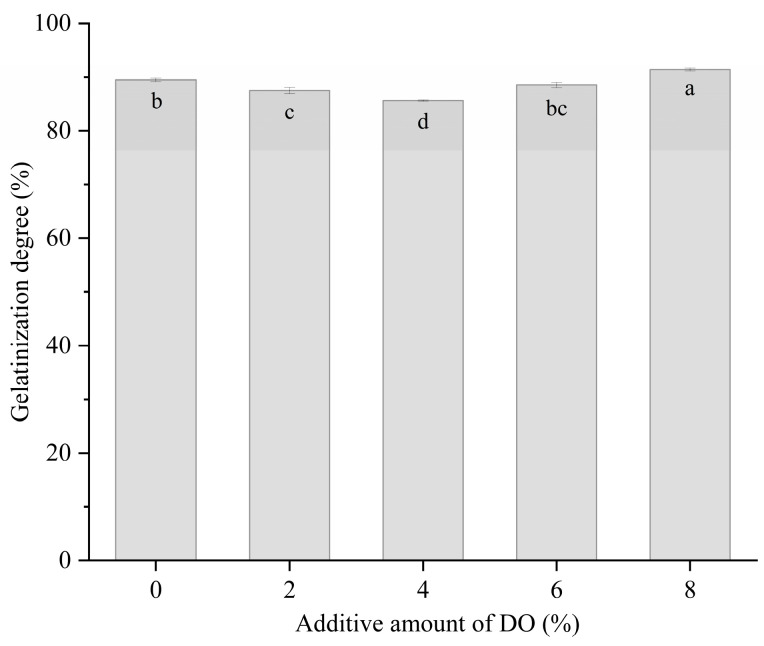
Gelatinization degree of cooked multi-grain noodles. DO, Dendrobium officinale. Different small letters in the graph indicate significant differences (*p* ≤ 0.05) among cooked noodles that differ in DO content.

**Figure 5:**
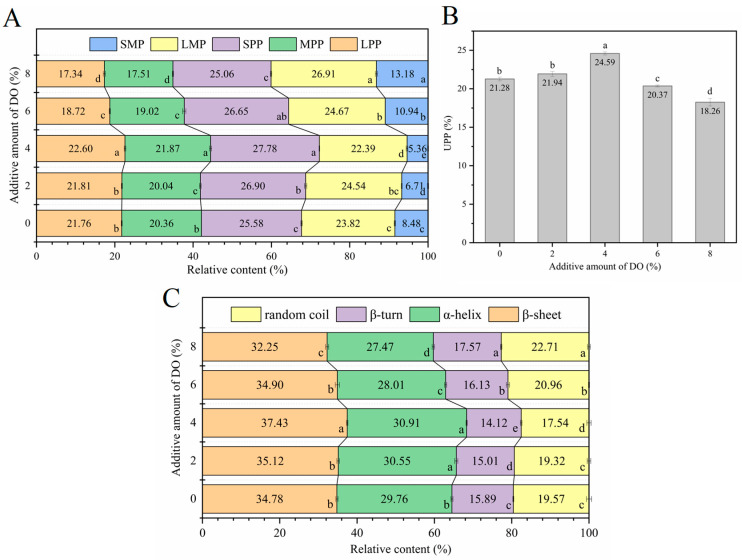
Protein behaviors of cooked multi-grain noodles that differ in DO content. (**A**) is the relative content of different protein fractions, (**B**) is the UPP content, and (**C**) is the relative content of different protein secondary structures. DO, Dendrobium officinale. Different small letters in the figure indicate significant differences (*p* ≤ 0.05) among the cooked noodles that differ in DO content.

**Figure 6:**
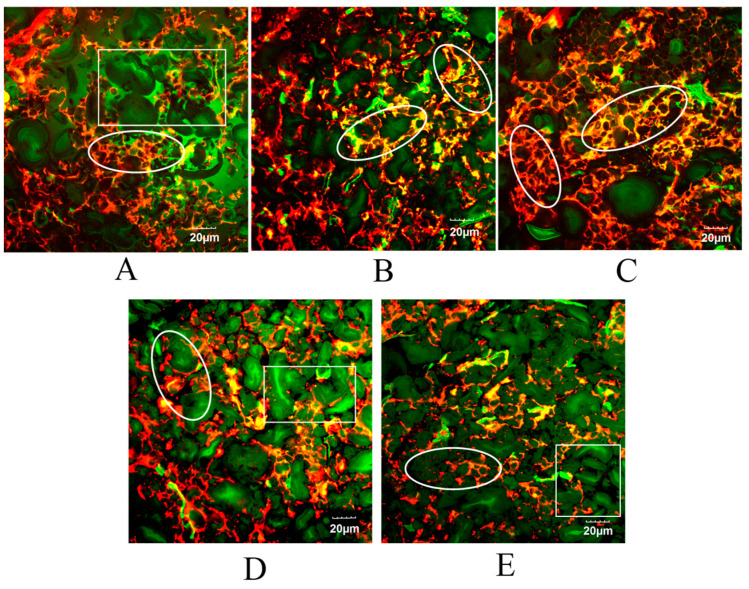
CLSM microstructures of cooked multi-grain noodles that differ in DO content. (**A**–**E**) are the cooked noodles containing 0%, 2%, 4%, 6%, and 8% DO, respectively. DO, Dendrobium officinale.

**Figure 7:**
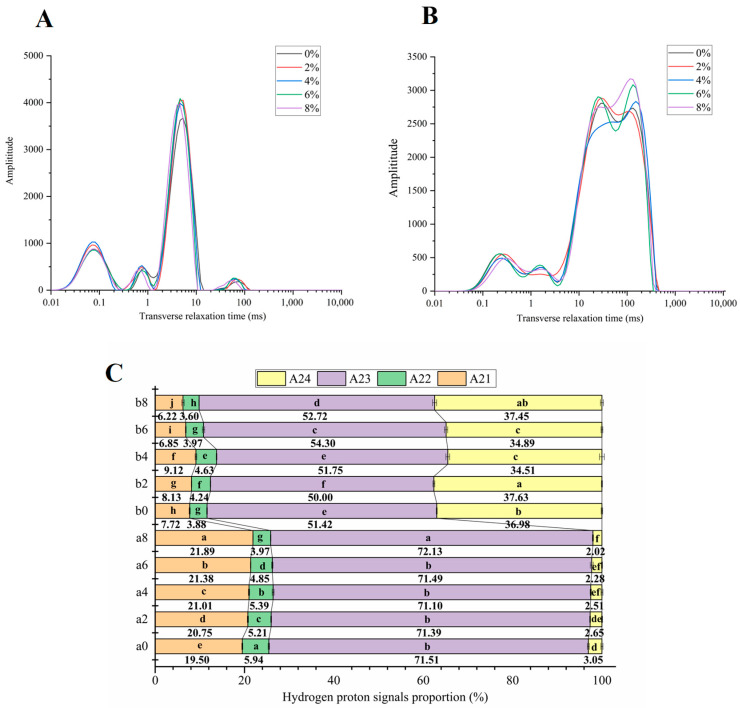
T2 spectrum of raw (**A**) and cooked (**B**) noodles and the relative areas of different proton populations in raw and cooked noodles (**C**), a0, a2, a4, a6, and a8 are raw noodles containing 0%, 2%, 4%, 6%, and 8% DO, respectively, and b0, b2, b4, b6, and b8 are cooked noodles containing 0%, 2%, 4%, 6%, and 8% DO added, respectively. DO, Dendrobium officinale. Different small letters in Figure (**C**) indicate significant differences (*p* ≤ 0.05) among the cooked noodles that differ in DO content.

The gelatinization degree of starch is defined as the percentage of gelatinized starch to the total starch content. During heat treatment, discontinuous gluten protein networks or weak starch–protein complexes allow significant amounts of water-soluble components, particularly amylose, to dissolve in hot water [37], resulting in a high degree of gelatinization. Figure 2 illustrates the effect of DO on the gelatinization degree of the cooked multi-grain noodles. The gelatinization degree decreased from 89.50% to 85.63% in the cooked noodles as DO increased from 0% to 4%. This change can be explained in two ways. First, low levels of DO (2–4%) enhanced the protein network surrounding the starch granules (Figure 5 and Figure 6), thereby inhibiting water absorption and the gelatinization of starch. Second, DO competes with starch for water absorption, which also contributes to the decrease in the gelatinization degree. The cooked noodles with 4% DO exhibited the lowest gelatinization degree of 85.63%, which may partially explain their maximum hardness (Figure 1). In contrast, the gelatinization degree of starch increased to 88.51% and 91.41% in the noodles containing 6% and 8% DO, respectively. This result can be attributed to further starch gelatinization in the noodles, promoted by the disruption of the gluten network (Figure 5 and Figure 6) and the migration of water from protein to starch (Figure 7).

#### 3.2.2. In Vitro Digestive Characteristics of Noodle Starch

**Figure 3:**
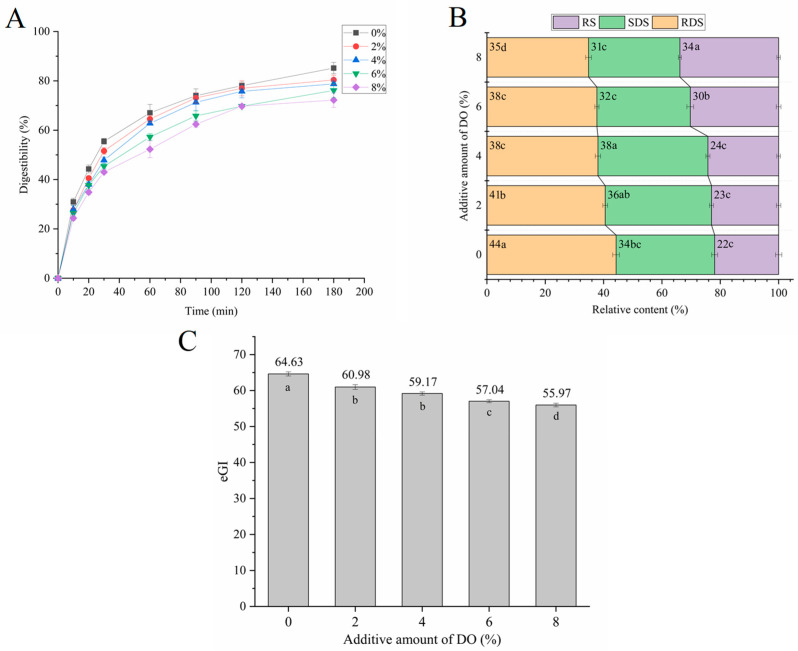
The digestion properties of starch from cooked multi-grain noodles that differ in DO content. (**A**) is the in vitro digestion curve of cooked noodles; (**B**) is the content of RDS, SDS, and RS in cooked noodles; (**C**) is the eGI values of cooked noodles. DO, Dendrobium officinale. Different small letters in the figure indicate significant differences (*p* ≤ 0.05) between cooked noodles that differ in DO content.

The digestion of noodle starch was inhibited by the increase in DO (Figure 3). The RDS content in the cooked noodles decreased continuously with the addition of DO. Conversely, the content of SDS increased significantly when DO rose from 0% to 4%. However, when the dosage of DO exceeded 4%, the SDS content decreased significantly, accompanied by a substantial increase in RS. This indicates that a portion of the SDS was converted to RS at higher levels of DO addition. Similar to the changes observed in RS, the eGI value of the noodles decreased continuously with increasing DO levels (Figure 3C), reaching a value of 55.97 when the noodles contained 8% DO. The enhanced starch digestion resistance in the cooked noodles is likely due to the increased adhesiveness imparted by DO (Figure 1B), which inhibits the diffusion of digestive enzymes, thereby reducing the digestibility of the noodles. Additionally, Dendrobium polysaccharide has the potential to enhance the macromolecular network surrounding the starch granules in the noodles, which may also limit water absorption, swelling, gelatinization, and enzymatic digestion of noodle starch [38,39] despite the dilution of the protein network.

#### 3.2.3. Order Degree and Helical Structure of Noodle Starch

**Figure 4:**
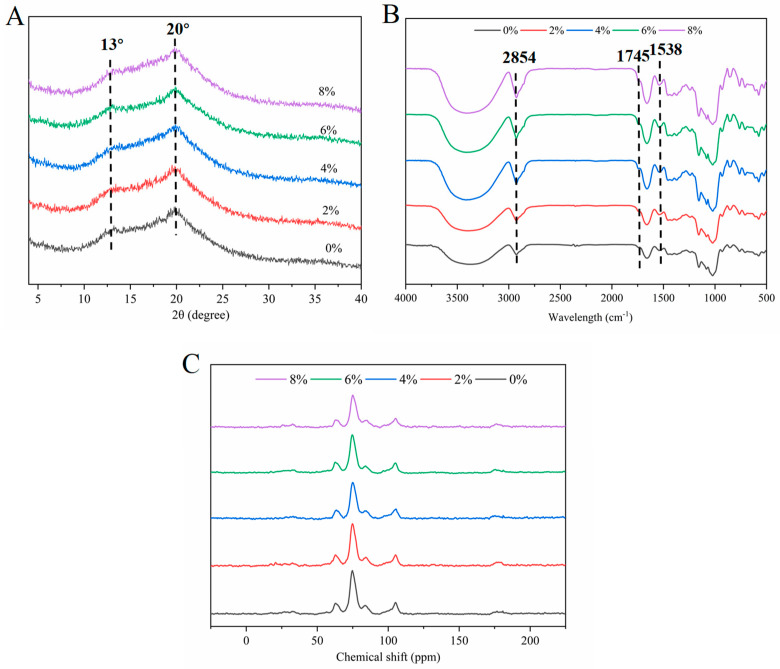
XRD (**A**), FTIR (**B**), and ^13^C NMR (**C**) patterns of cooked multi-grain noodles. DO, Dendrobium officinale.

Figure 4A–C present the XRD diffraction patterns, FTIR spectra, and ^13^C NMR spectra of the cooked noodles, respectively. As illustrated in Figure 4A, the starch in the cooked noodles exhibits a distinct V-type crystal structure, characterized by a peak at 20°. The diffraction peak at 13° is associated with the complexes formed between starch and small molecules [40], indicating that the gelatinized starch has interacted with proteins or lipids, which facilitates the formation of V-type crystals [24]. In the case of the cooked noodles, the addition of DO at lower concentrations (2% and 4%) significantly increased the relative crystallinity of the noodle starch (Table 4). However, further increases in the proportion of DO (6–8%) resulted in a decrease in the relative crystallinity of noodle starch. This finding suggests that a relatively lower content of Dendrobium polysaccharide promotes the formation of V-type crystals and an ordered starch structure in cooked noodles, thereby enhancing the relative crystallinity of gelatinized starch. Conversely, higher concentrations of DO (6% and 8%) tend to dilute the protein network (Figure 6) and encourage water absorption, swelling, and cracking of starch during the cooking process, ultimately leading to a reduction in the relative crystallinity of noodle starch (Table 4).

It is well known that the FTIR spectrum at 995/1022 (DD value) is positively correlated with the double helicity of starch, while the spectrum at 1047/1022 (DO value) is positively correlated with the short-range order of starch [40,41]. As shown in Table 4, the cooked noodles containing 4% and 8% Dendrobium powder exhibited higher DD values and enhanced short-range order of starch, indicating an improved short-range order. This finding is also consistent with the significantly stronger peak intensity observed between 3000 and 3600 cm^−1^, which is positively related to the strength of hydrogen bonds in starch (Figure 4B).

The FTIR peak at 1538 cm^−1^ indicates starch–protein complexes, while peaks at 2854 cm^−1^ and 1714 cm^−1^ indicate starch–lipid complexes. Their simultaneous presence suggests starch–lipid–protein terpolymers [42]. Figure 4B shows that these peaks were present in all the cooked noodles, with the intensity increasing as DO increased from 0% to 8%. This indicates that DO enhances the formation of protein–starch, protein–lipid, or starch–lipid–protein complexes, with the quantity of complexes positively correlated to DO levels.

The long- and short-range molecular order of starch, along with the interactions between starch and protein/lipid in various cooked noodles, suggests that the optimal textural, cooking, and sensory performance of the noodles with 4% DO can be primarily attributed to the superior long- and short-range order of gelatinized starch. However, with a further proportional increase in DO, the resistance to starch digestion is likely enhanced by both the high adhesiveness of the noodles (Figure 1A) and the strong interactions between starch and protein/lipid.

Nuclear magnetic resonance spectroscopy was employed to quantify the short-range ordered helical structures in the starch in the cooked noodles, aiming to elucidate the changes in single helix, double helix, and amorphous structures, as well as the detailed conformational transformations of noodle starch [43]. Figure 4C presents the ^13^C NMR diffraction pattern of the cooked noodles, where the wide peaks at 103 or 104 ppm are associated with typical single helical structures found in V-type crystalline regions or dispersed within amorphous regions. In contrast, peaks near 76 ppm are positively correlated with the double helix content in starch [44]. The content of single and double helices in the starch from the cooked noodles significantly increased with the rise in DO from 0% to 4% (Table 4). Given the consistent upward trend in both long- and short-range order of noodle starch (Table 4), we propose that lower DO levels promote the formation of a more stable macromolecular network structure. This stability reduces the degree of starch damage and enhances its orderliness during the cooking process. Conversely, further increases in DO (6–8%) levels resulted in a decrease in the content of double helices, despite an increase in the proportions of single helices and the amorphous region. The finding suggests that the high DO levels convert double helices in starch into single helices, which aligns with the observed low relative crystallinity and high short-range order of starch in the cooked noodles with elevated DO levels (6–8%). Notably, the cooked noodles supplemented with 8% DO exhibited the highest single helix content and short-range molecular order (Table 4) among all the samples. This phenomenon may be attributed to the unraveling of starch chains and the formation of ordered starch–protein–lipid complexes during the cooking process [45].

### 3.3. Polymerization Degree and Secondary Structure of Proteins in the Cooked Noodles

Based on the method described by Peng et al. [24], the SDS-extractable and unextractable proteins determined by SE-HPLC were categorized into five fractions according to their molecular weight: Larger Protein Polymers (LPPs), Medium Protein Polymers (MPPs), Smaller Protein Polymers (SPPs), Larger Monomer proteins (LMPs), and Smaller Monomer Proteins (SMPs). The proportion of SDS-unextractable LPPs and MPPs relative to the total amount of SDS-extractable and unextractable polymeric proteins was defined as the percentage of Unextractable Protein Polymers (UPPs).

The content of polymeric proteins (LPPs, MPPs, and SPPs) increased proportionally with the rise in DO from 0% to 4% (Figure 5A), indicating that the protein networks in the cooked noodles were extended and enhanced with the addition of DO at the lower levels. However, when the DO concentration exceeded 4%, the levels of polymeric proteins decreased as the content of monomeric proteins increased. Notably, the increase in SMPs was greater than that of LMPs, suggesting that higher percentages of DO led to the disruption of the protein network in the cooked noodles. As illustrated in Figure 5B, the UPP% initially increased and then decreased with the rise in DO content, with the highest UPP content (24.59%) observed in the cooked noodles containing 4% DO. This phenomenon demonstrates that low supplemental levels (2–4%) of DO are conducive to the formation of a stable and dense protein network in the cooked noodles, thereby contributing to the optimal noodle quality. Conversely, high supplemental levels (6–8%) of DO weakened the protein network, potentially promoting starch–protein interactions and resulting in increased digestion resistance of noodle starch.

Figure 5C illustrates the relative proportion of various secondary structures in noodle proteins. A significant increase in β-sheet and α-helix content, along with a decrease in β-turn and random coil structures, was observed in noodle proteins as the percentage of DO increased from 0% to 4%. However, further increases in DO resulted in the opposite trends for all the secondary structures. Since β-sheets and α-helices are positively correlated with the compactness of protein structures [46,47], while β-turns and random coils are typically associated with disordered and loose protein structures [48], these changes align with the protein behaviors detected by SE-HPLC (Figure 5A). Specifically, lower percentages of DO (2–4%) facilitated the formation of a compact and extended protein network in the cooked noodles, whereas a higher percentage of DO led to a weak and loose protein network. In summary, the low supplemental levels of DO (0–4%) promoted the development of an extensive and compact protein network in the cooked noodles, as evidenced by the high content of polymeric proteins and UPP rich in β-sheets and α-helices, which significantly improved noodle quality. In contrast, the excessive amounts of DO (6% and 8%) resulted in a poor protein network characterized by a high content of monomeric proteins rich in β-turns and random coils, which adversely affected starch–protein interactions and starch digestion resistance in the noodles.

### 3.4. Microstructure Characteristics of the Cooked Noodles

Figure 6 illustrates the cross-sectional microstructure of the cooked noodles as observed by CLSM. In this figure, the green and red areas represent starch and protein, respectively. The starch and gluten networks in noodles are expected to undergo complex changes due to the swelling and rupture of starch granules, as well as the depolymerization and reconstruction of the protein network during cooking [49]. The cooked noodles without DO exhibited the largest gelatinized starch clusters (marked in the box in Figure 6A) and discontinuous protein aggregates surrounding the large starch clumps. This observation is consistent with a higher degree of starch gelatinization (Figure 2) and lower protein polymerization (Figure 5). In contrast, the swelling and destruction of starch granules were inhibited in the cooked noodles containing 2% DO, resulting in smaller starch clumps enveloped by a relatively continuous protein network (marked in the circle of Figure 6B). Furthermore, the most extensive and compact protein network that tightly encased the partially gelatinized starch granules was observed in the cooked noodles with 4% DO (marked in the circle of Figure 6C), which correlates with the highest UPP% and optimal quality performance.

In the cooked noodles supplemented with 6% and 8% DO, a great number of gelatinized starch granules were closely interconnected after swelling, resulting in the formation of larger starch clusters (as indicated in the boxes of Figure 6D,E). Additionally, the protein network structure was disrupted into filamentous fragments (as shown in the circles of Figure 6D,E), significantly diminishing its coating effect on starch. It appears that the excessive amount of DO facilitated the gelatinization, aggregation, and further adhesion of starch granules, thereby enhancing the adhesiveness and starch digestion resistance of the cooked noodles.

The aforementioned differences suggest that the addition of DO at lower levels (2% and 4%) can enhance the degree of polymerization and the compactness of the protein network in noodles. This improvement, in turn, enhances the ability of noodles to encapsulate starch granules, thereby improving the overall quality of noodles. However, a relatively high concentration of DO may lead to the disruption of the protein network and the dissolution of starch granules. Increased levels of DO in cooked noodles can enhance the adhesion between gelatinized starch granules and the interactions of starch with non-starch components, resulting in significant increases in adhesiveness and starch digestion resistance in cooked noodles.

### 3.5. Particle Size Distribution of the Cooked Noodles

The D10, D50, and D90 values of the lyophilized powder of the cooked noodles represent the particle sizes at which 10%, 50%, and 90% of the noodle particles are cumulatively distributed within the system. D50 is commonly used to indicate the average particle size of flour [50]. As shown in Table 5, the D50 of the cooked noodles increased continuously from 32.83 µm to 47.85 µm with the increasing addition of DO from 0% to 8%. This trend is consistent with the aggregation behavior of gelatinized starch observed in Figure 6. The smallest particle size was found in the cooked noodles without DO, suggesting that the fully gelatinized starch granules, lacking the support of an extensive gluten network or adhesion from DO (marked by boxes in Figure 6D,E), were more prone to breaking down into much smaller particles after freeze-drying and grinding. When the DO concentration was 2% or 4%, the partially gelatinized starches were enveloped by a dense and extensive protein network (Figure 6B,C), leading to a continuous protein–starch macromolecular structure in the cooked noodles that promoted the formation of larger particle sizes. A higher content of DO (6% or 8%) resulted in a significant increase in the particle size of the cooked noodles, despite the serious disruption of the protein network (Figure 6D,E). This indicates that the aggregation of the gelatinized starch was primarily facilitated by the adhesive effect of DO, ultimately contributing to a more solid and compact structure and larger particle size in the cooked noodles.

The granule size is a critical factor in determining the digestibility of raw starch due to variations in viscosity and resistance to enzymatic hydrolysis [51,52]. Noodles characterized by a loose structure and small particle size possess a larger specific surface area and more potential enzyme binding sites, which facilitate rapid enzymatic hydrolysis [53]. This may explain why the starch digestion resistance of the cooked noodles is positively correlated with the amount of DO.

### 3.6. Water Distribution of the Noodles

The transverse relaxation time (T2) profiles of the raw and cooked noodles are present in Figure 7A and Figure 7B, respectively, while Figure 7C illustrates the relative areas of different proton populations in both the raw and cooked noodles. Four distinct proton populations are identified in Figure 7A,B, labeled A21, A22, A23, and A24, based on their varying T2 values. According to previous studies, population A21 primarily consists of CH protons from immobile crystallized amylose and/or amorphous starch, as well as rigid protons from gluten. Populations A22 and A23 comprise CH protons from amorphous starch and gluten located in sheets that are in contact with confined water. In contrast, population A24 is associated with mobile protons of water that exchange with hydroxyl protons of starch on the granule surface, as well as water protons surrounding the sheets that exchange with gluten protons [54].

As shown in Figure 7A, the addition of DO did not induce significant changes in the T2 relaxation time of different proton populations in the fresh noodles; however, notable differences in the relative peak area among various fresh noodle samples were observed (Figure 7C). Specifically, as the proportion of DO in the fresh noodles increased, the A21 signal rose continuously, while the A22 and A24 signals correspondingly decreased. In contrast, A23 showed minimal sensitivity to lower levels of DO, with a significant increase only observed in the noodles containing 8% DO. This phenomenon suggests that the addition of DO inhibited the proton mobility in the fresh noodles, and this effect was dose-dependent. DO is likely to interact with starch granules and the gluten network, impeding the interactions between confined water and protons of amorphous starch or gluten sheets. This interaction resulted in an increasing proportion of rigid protons in noodle starch and gluten (A21). Additionally, the low proportion of A24 and its continuous decrease with increasing DO content can be attributed to both the reduced water content in the fresh noodles and the strong water-holding capacity of DO, which together limit the proton exchange between free water and starch/gluten.

The four peaks of all the cooked noodles shift toward longer T2 values when compared to their fresh counterparts, indicating that starch gelatinization and gluten denaturation and reconstruction, induced by water absorption and heating, significantly increased the mobility of all proton populations. In comparison to the fresh noodles, the cooked noodles exhibited a much lower intensity of A21, A22, and A23, while A24 was found to be higher (Figure 7B,C). This change was partly attributed to the high water absorption of the cooked noodles (Table 2), which significantly increased the relative amount of free water, thereby decreasing the proportion of bound water. Additionally, some confined water trapped within the starch granules and gluten network of the fresh noodles was more likely to be exposed and merge with external water during the cooking process, further promoting the proportional increase in free water. Notably, the cooked noodles containing 4% DO exhibited the highest A21 and A22 values, along with the lowest A24 among all the cooked noodles. This is likely due to the optimal protein network present in this formulation (Figure 5 and Figure 6). We believe that the addition of 4% DO enhanced water absorption, as well as the denaturation and reconstruction of the protein network. This dense and compact macromolecular network, filled with partially gelatinized starch, is thought to trap more water, resulting in the conversion of A24 to A21 and A22 [29]. In contrast, the higher percentage of DO (6% and 8%) tended to dilute and disrupt the dense and extensive protein network formed in the cooked noodles with 4% DO, thereby converting the rigid water (A21 and A22) trapped in the thermoset gluten into mobile water (A23 and A24). These mobile waters, resulting from the disrupted protein network, appear to be absorbed by starch and DO, promoting their further gelation and complexation (Figure 2 and Figure 6). This process ultimately increased the adhesiveness (Figure 7B) and starch digestion resistance of the cooked noodles with a higher percentage of DO (Figure 3), although their sensory quality was diminished.

## 4. Conclusions

The molecular behaviors during noodle cooking, including protein network formation or destruction, starch gelatinization, interactions between starch and non-starch components, and the water migration between starch and protein, were closely dependent on the amount of DO in the noodles. Lower supplemental levels of DO (2% and 4%) facilitated the protein hydration and formation of a more compact and extensive protein network that enveloped the partially gelatinized starch with higher relative crystallinity. However, higher proportions of DO (6% and 8%) diluted and disrupted the protein network in the cooked noodles, resulting in the release of significant amounts of protein-bound water, which participated in the co-gelatinization of starch and DO polysaccharides. The findings of this study provide a reference for regulating the sensory quality or starch digestibility of multi-grain noodles through the addition of an appropriate amount of DO. The observed increase in starch digestion resistance was attributed to both the aggregation of gelatinized starch and the interactions between starch and non-starch components. Further research is needed to quantify the degree of contribution from these two factors. Generally, incorporating an appropriate amount of DO in the preparation of traditional wheat or multi-grain noodles has the potential to develop innovative noodle products with optimal sensory and nutritional qualities. This approach also contributes to expanding the application of medicinal and edible plants. However, the exact influence of DO polysaccharides on noodle quality was not fully clarified in this study. Therefore, future research should explore the combination of polysaccharide extracts from DO with cereal starch, protein, or their mixtures for noodle preparation in order to elucidate the mechanisms by which DO polysaccharides affect the quality and digestion properties of noodles.

## Figures and Tables

**Table 1:** Chemical composition of flours and Dendrobium officinale.

Sample	Total Starch (%)	Protein (%)	Fat (%)	Dietary Fiber (%)	Polyphenol (%)	Flavone (%)
Wheat flour	73.30 ± 2.11 a	11.20 ± 1.81 b	1.20 ± 0.89 c	3.17 ± 0.24 d	0.14 ± 0.31 c	0.16 ± 0.29 c
Common buckwheat flour	48.74 ± 0.01 c	14.17 ± 0.00 a	3.67 ± 0.19 b	5.45 ± 0.00 c	0.52 ± 0.11 a	1.09 ± 0.02 a
Oat flour	59.85 ± 1.02 b	13.20 ± 0.56 ab	5.92 ± 0.81 a	8.26 ± 0.56 b	0.06 ± 0.23 d	0.14 ± 0.17 c
Dendrobium officinale	9.45 ± 0.21 d	5.99 ± 0.04 c	1.15 ± 0.01 c	59.81 ± 0.52 a	0.35 ± 0.17 b	0.19 ± 0.11 b

Means with different small letters within the same column are significantly different at *p* < 0.05. DO: Dendrobium officinale.

**Table 2:** Cooking quality of multi-grain noodles.

Additive Amount of DO (%)	Cooking Time (min)	Water Absorption (%)	Cooking Loss (%)
0	3.00	67.42 ± 1.26 e	17.68 ± 0.86 a
2	3.25	80.78 ± 0.91 d	15.42 ± 1.26 b
4	3.25	83.73 ± 1.07 c	13.75 ± 0.44 c
6	3. 5	85.48 ± 0.86 b	12.89 ± 3.11 c
8	3.75	87.33 ± 0.59 a	9.25 ± 2.28 d

Means with different small letters within the same column are significantly different at *p* < 0.05. DO: Dendrobium officinale.

**Table 3:** Sensory quality of multi-grain noodles.

Additive Amount of DO (%)	Color and Luster	Apparent State	Oral (Soft and Hard)	Toughness	Stickiness	Smoothness	Palatability	Total Points
0	8.5 ± 0.4 a	8.6 ± 0.5 a	14.8 ± 0.4 c	16.9 ± 0.6 d	16.0 ± 0.4 c	3.6 ± 1.1 c	4.5 ± 0.7 a	73.2 ± 0.6 c
2	8.0 ± 0.7 b	8.0 ± 0.8 b	16.7 ± 0.5 b	18.1 ± 0.5 b	16.1 ± 1.0 c	3.7 ± 0.4 c	4.0 ± 1.0 b	74.6 ± 0.72 b
4	7.8 ± 1.0 b	7.5 ± 0.7 c	17.4 ± 0.5 a	20.0 ± 0.6 a	16.9 ± 0.6 b	4.0 ± 0.7 b	3.8 ± 0.9 c	77.4 ± 0.62 a
6	7.5 ± 0.8 c	7.1 ± 0.5 d	15.0 ± 0.3 c	17.4 ± 0.4 c	17.2 ± 0.7 b	4.6 ± 0.8 a	3.6 ± 0.4 c	72.3 ± 0.51 c
8	7.4 ± 0.5 bc	7.0 ± 0.8 d	13.3 ± 0.6 d	16.5 ± 0.3 d	18.5 ± 0.4 a	4.5 ± 0.6 a	3.1 ± 0.3 d	70.3 ± 0.97 d

Means with different small letters within the same column are significantly different at *p* < 0.05. DO: Dendrobium officinale.

**Table 4:** Orderliness and carbon chain structure of starch in cooked multi-grain noodles.

Additive Amount of Dendrobium Officinale (%)	Long Range Order Degree by XRD	Short-Range Order Degree by FTIR		Double Helix (%)	Single Helix (%)	Amorphous Region (%)
	The Relative Crystallinity (%)	DD (995/1022 cm^−1^)	DO (1047/1022 cm^−1^)			
0	12.79 ± 0.21 c	1.0651 ± 0.0022 c	1.0827 ± 0.0036 e	53.62 ± 0.43 c	0.71 ± 0.07 d	9.57 ± 0.29 c
2	14.30 ± 0.39 b	1.0782 ± 0.0017 c	1.1005 ± 0.0004 d	55.45 ± 1.01 b	0.86 ± 0.08 cd	8.03 ± 0.57 d
4	15.76 ± 0.68 a	1.2372 ± 0.0020 ab	1.2266 ± 0.0030 b	57.36 ± 0.70 a	0.98 ± 0.05 c	9.24 ± 0.70 c
6	13.03 ± 0.24 c	1.2019 ± 0.0034 b	1.2073 ± 0.0044 c	52.47 ± 0.65 cd	1.31 ± 0.22 b	10.62 ± 0.25 b
8	11.06 ± 0.46 d	1.2638 ± 0.0034 a	1.2431 ± 0.0038 a	51.64 ± 0.83 d	1.98 ± 0.15 a	12.31 ± 0.62 a

Means with different small letters within the same column are significantly different at *p* < 0.05.

**Table 5:** Particle size distribution of cooked multi-grain noodles.

Additive Amount of DO (%)	D10 (µm)	D50 (µm)	D90 (µm)
0	6.64 ± 0.30 d	32.83 ± 0.25 e	159.60 ± 0.19 e
2	7.71 ± 0.15 c	37.13 ± 0.35 d	172.00 ± 0.08 d
4	7.91 ± 0.25 c	39.68 ± 0.42 c	180.15 ± 0.19 c
6	8.36 ± 0.07 b	44.17 ± 0.18 b	190.90 ± 1.13 b
8	8.67 ± 0.16 a	47.85 ± 0.48 a	217.80 ± 0.09 a

Means with different small letters within the same column are significantly different at *p* < 0.05. DO: Dendrobium officinale.

## Data Availability

The authors do not have permission to share data.

## Supplementary Materials

The following supporting information can be downloaded at: https://www.mdpi.com/article/10.3390/foods14030413/s1, Supplementary Table S1: Testing items and scoring criteria for sensory evaluation of noodles.
