# Supplementary material for: The Quality and Starch Digestibility of Multi-Grain Noodles Are Regulated by the Additive Amount of Dendrobium Officinale
Source: Foods. 2025 Jan 27;14(3):413. doi: 10.3390/foods14030413 (PMC11817478; doi:10.3390/foods14030413)
Supplement: Supplementary file 1 [file foods-14-00413-s001.zip › foods-3385028-supplementary.pdf]

Table. S1 Testing items and scoring criteria for sensory evaluation of noodles

Item	Full marks	Scoring standard
Color and luster	10	The color and brightness of noodle<br>White, milky, milky yellow, brightness: 8.5~10<br>Medium brightness: 6~8.4<br>Dark, gray, low brightness: 1~6
Apparent state	10	The degree of smoothness and expansion of the noodle surface<br>The surface structure is fine and smooth: 8.5~10<br>The middle: 6.0~8.4<br>The surface is rough, expansion, and serious deformation:1~6
Oral (soft and hard)	20	The force required to cut up a piece of noodle with teeth.<br>Moderate hardness: 17~20<br>Slightly hard or soft: 12~17<br>Too hard or too soft: 1~12
Toughness	25	The elasticity of the noodle during chewing<br>Good elasticity: 21~25<br>Medium elasticity: 15~21<br>Insufficient elasticity: 1~15
Stickiness	25	The sticking strength of noodle during chewing<br>Refreshing and non-sticky: 21~25<br>Slightly refreshing and medium stickiness' 15~21<br>Non-refreshing and sticky: 10~15
Smoothness	5	The smoothness of noodle<br>Smooth: 4.3~5<br>Medium smoothness: 3~4.3<br>Poor smoothness: 1~3
Palatability	5	The taste of noodle<br>Fresh fragrance 4.3~5<br>Odorless: 3~4.3<br>Peculiar smell: 1~3
Total points	100	During the evaluation, pure wheat noodle was used as a standard noodle with the score of 100
